# Three-dimensional object-based quantification of nicotinic acetylcholine receptor subunit colocalization using Imaris

**DOI:** 10.64898/2026.10.11.758376

**Published:** 2026-10-19

**Authors:** Ankura Sitaula, Aref Zarin

## Abstract

Neural circuit function depends not only on synaptic connectivity but also on the molecular composition and spatial organization of neurotransmitter receptors. Determining whether receptor subunits occupy shared or distinct domains requires spatial measurements capable of resolving sparse receptor puncta across neural tissue and, when needed, restricting analysis to defined cellular compartments. Conventional intensity-based colocalization metrics can be difficult to interpret for sparse, punctate signals and do not inherently provide object-level classification or cell-specific analysis. Here, we describe a three-dimensional object-based colocalization workflow for endogenously tagged nicotinic acetylcholine receptor (nAChR) subunits imaged by Airyscan super-resolution confocal microscopy and analyzed in Imaris. Individual receptor puncta are segmented as 3D surface objects and classified as colocalized or non-colocalized using a control-defined spatial overlap criterion. The workflow supports analysis within a defined region of the ventral nerve cord neuropil or within genetically labeled motor-neuron dendritic compartments. For dendrite-restricted analysis, receptor puncta are first selected by their overlap with the dendritic surface before receptor–receptor overlap is quantified. The overlap criterion is empirically calibrated using positive and negative biological controls processed under the same imaging and analysis conditions, providing a reproducible alternative to arbitrary threshold selection. This approach enables receptor organization to be quantified within the neuropil and genetically labeled motor-neuron dendritic compartments and can be adapted to other epitope-tagged receptors, ion channels, and synaptic proteins.

**Key features:**

- Enables 3D object-based colocalization analysis of sparse, punctate receptor signals that may be difficult to evaluate using conventional intensity-based approaches.
- Supports colocalization analysis at two spatial scales: within defined neural tissue regions and within genetically labeled neuronal dendritic compartments.
- Particularly suited for endogenously tagged receptors and other punctate membrane proteins imaged by high-resolution confocal or Airyscan microscopy.
- Uses experimentally validated positive and negative controls to establish overlap criteria for classifying individual 3D puncta as colocalized or non-colocalized.

**This protocol is used in:**

PNAS Nexus (2026), DOI: https://doi.org/10.1093/pnasnexus/pgag173

**Graphical overview:**

**
Graphical overview. 3D object-based workflow for analyzing nAChR subunit colocalization in the *Drosophila* larval ventral nerve cord** . (A) Dissect and immunostain third-instar larvae, then acquire a three-dimensional (3D) Airyscan image stack of the selected ventral nerve cord (VNC) region. (B) Segment the fluorescence signals of receptor subunits X and Y as separate 3D surfaces in Imaris. (C) Define the overlap criterion using a positive control (dual -tagged α6::HA and α6::OLLAS; >98% overlap) and a negative control (Rdl::HA and α5::OLLAS; <2% overlap). Classify objects as overlapping when the *Overlapped Volume to Surfaces* value is ≥0.05 µm ^3^ . (D) Assess receptor overlap using one of two analysis regions: the dorsal VNC neuropil or motor-neuron (MN) dendrites. For the MN dendrite-restricted analysis, first retain the surfaces of each receptor subunit that overlap the labeled dendrites, then assess overlap between the two retained receptor populations. (E) Count overlapping (CoLo) and non-overlapping (N.CoLo) receptor objects. The bar graph illustrates the two output categories; its values do not represent experimental data.

